# Geometric effects of volume-to-surface mapping of fMRI data

**DOI:** 10.1007/s00429-022-02536-4

**Published:** 2022-07-27

**Authors:** Keith George Ciantar, Christine Farrugia, Paola Galdi, Kenneth Scerri, Ting Xu, Claude J. Bajada

**Affiliations:** 1 Department of Physiology and Biochemistry, Faculty of Medicine and Surgery, L-Università ta’ Malta, Msida, MSD 2080 Malta; 2 MRC Centre for Reproductive Health, The University of Edinburgh, Edinburgh, EH16 4TJ UK; 3 Department of Systems and Control Engineering, Faculty of Engineering, L-Università ta’ Malta, Msida, MSD 2080 Malta; 4 Center for the Developing Brain, Child Mind Institute, New York, NY 10022 USA

**Keywords:** Functional MRI, Volume-to-surface mapping, Artificial correlations, Cortical organization

## Abstract

In this work, we identify a problem with the process of volume-to-surface mapping of functional Magnetic Resonance Imaging (fMRI) data that emerges in local connectivity analysis. We show that neighborhood correlations on the surface of the brain vary spatially with the gyral structure, even when the underlying volumetric data are uncorrelated noise. This could potentially have impacted studies focusing upon local neighborhood connectivity. We explore the effects of this anomaly across varying data resolutions and surface mesh densities, and propose several measures to mitigate these unwanted effects.

## Introduction

Taking a surface-based approach has become a popular choice when analysing fMRI data. Indeed, an increasing number of large projects provide surface-based data (e.g. Human Connectome Project [HCP] (Van Essen et al. 2012, 2013), Adolescent Brain Cognitive Development [ABCD] study (Bjork et al. 2017)), as well as software packages that facilitate surface analysis (e.g. FreeSurfer or Connectome Workbench). Recent fMRI packages have also implemented surface-based analysis in the preprocessing pipelines (e.g. the HCP pipelines (Glasser et al. 2013) and fMRIPrep (Esteban et al. 2019)). There are good theoretical (Brodoehl et al. 2020; Glasser et al. 2016) and practical advantages (Coalson et al. 2018) to performing fMRI analysis on a cortical surface mesh. The primary advantages are that smoothing on a two-dimensional surface (as opposed to volume-based smoothing) respects the geometry of the brain and reduces signal contamination from cortical areas whose Euclidean separation is much smaller than the geodesic distance between them—such as the opposing banks of a sulcus. Additionally, in studies across different subjects, surface registration has been shown to give better alignment of cortical landmarks in comparison to volume-based registration (Anticevic et al. 2008; Ghosh et al. 2010); it may also enhance the statistical power profiles of cortical fMRI activation data that have undergone little to no smoothing (Anticevic et al. 2008), increasing the sensitivity of the statistical analysis to activated regions (Tucholka et al. 2012). Spatial smoothing on the surface manifold can moreover improve signal resolution and preservation relative to a comparable amount of smoothing of volumetric data (Anticevic et al. 2008).

As part of the surface-based preprocessing pipeline, the user must first map their volumetric (voxel) data to surface vertices. This has the potential of introducing artefacts that may affect downstream surface-based analyses. In this work, we identify and report a problem with the process of volume-to-surface mapping in the context of local neighborhood connectivity analysis. The effect has potentially impacted studies that have not taken it into consideration.

## The problem

**Fig. 1:**
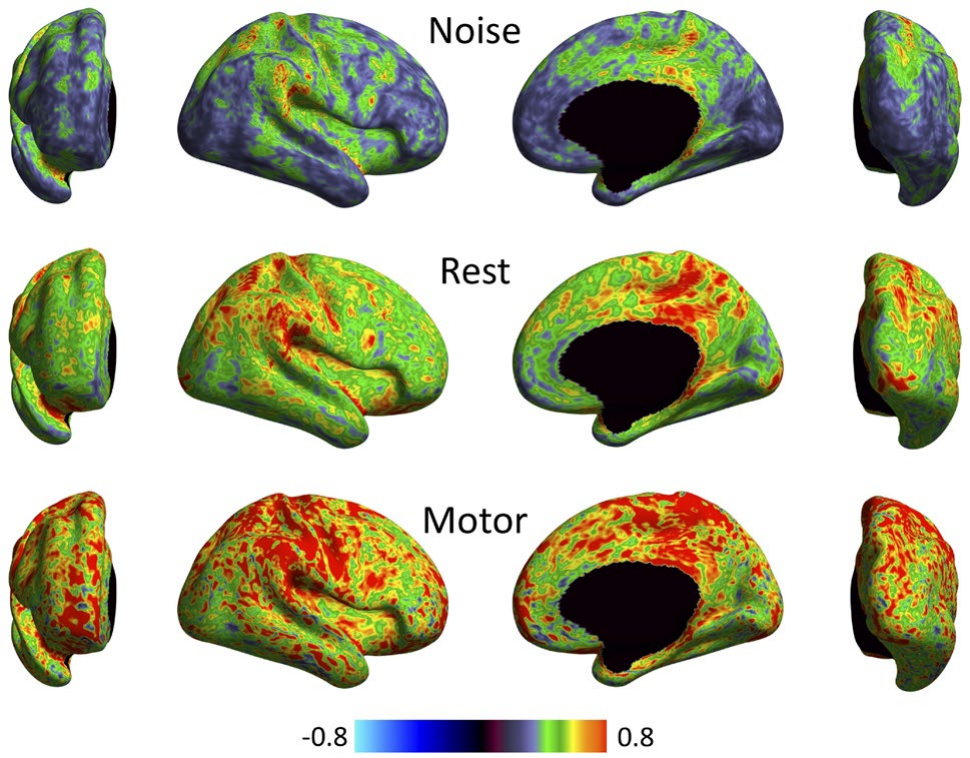
A delineation of the observed anomaly. The presence of local correlations that mirror the pattern of gyral folds emerges clearly, even in the case of noise. The figure shows surface-based, vertex-wise neighborhood correlation maps for three separate sets of volumetric data: noise (top row), resting-state (middle) and task data (bottom). Volume-to-surface mapping was carried out using the direct approach (whereby the data are mapped directly to the target surface mesh, and no up/down sampling is involved) with cubic interpolation. The reader is cautioned that if temporal autocorrelation is factored in (Afyouni et al. 2019), maps obtained with different sets of data would not remain directly comparable. However, the general pattern of correlations would not change

An anomaly was noted when analysing regional boundaries (or edges) in functional signatures across the cortex (Bajada et al. 2020). Specifically, when aiming to characterize regional boundaries by calculating functional changes between neighboring vertices along the surface, we observed a clear anatomical pattern that follows the gyral folds of the brain, even when using stochastic data as input (Fig. 1). This suggests that the local connectivity analysis might be affected, at least partially, by surface geometry.

A related phenomenon was first noted in (Glasser et al. 2013). The authors observed that ‘when mapping fMRI data of a given resolution to the midthickness surface, larger voxels can artificially increase the correlation between geodesically distant surface vertices, as larger voxels can span both sides of a sulcus or a thin gyral blade of white matter’. This explains why geometric artefacts tend to follow gyral/sulcal patterns. They furthermore noted that increasing the resolution of fMRI data decreases geometric effects on surface data. Their primary concern was, however, the implications this had for the correlation between relatively (geodesically) distant cortical areas.

In order to explore the phenomenon in the context of neighborhood correlations more rigorously, we used uncorrelated noise (drawn from a standard normal distribution) to create synthetic fMRI timeseries at the voxel level. These were then projected onto the surface using various volume-to-surface mapping approaches.

A measure of local connectivity was obtained by calculating the pairwise Pearson correlation between any two vertices within a local region (where a *local region* is defined as consisting of a given vertex and its neighborhood i.e. any vertices directly adjacent to it). Next, we performed the Fisher-z transform, and adopted the mean of* z* scores as the local connectivity index for the given vertex.

The presence of regional boundaries that follow gyral patterns emerged when using both real and stochastic data. This prompted a more systematic exploration of the issue, with the aim of understanding and mitigating the problem.

## Methodology

Our analysis makes use of cortical surfaces constructed from T1-weighted MRI scans, together with resting-state and task fMRI data, all sourced from the minimally preprocessed Human Connectome Project Young Adult data set (Glasser et al. 2013) (more information is provided in the Data Availability Section at the end of this manuscript). These data were selected for the high quality of their acquisition, as well as to ensure state-of-the-art preprocessing (up to the point where the discussed issue is encountered); we considered a single subject. Noise timeseries were generated at three isotropic resolutions (0.7 mm, 1.4 mm and 2 mm) in volumetric space and pushed to the surface using five different volume-to-surface algorithms available with Connectome Workbench (Marcus et al. 2011).^1^ These algorithms provide a way to associate volumetric data with the vertices on the midthickness surface mesh. The Enclosing method, for instance, simply assigns to each vertex the data of the voxel inside which it lies. The Trilinear approach involves doing a linear interpolation of the data from the eight voxels closest to the vertex, and the Cubic algorithm employs cubic splines. As explained in the documentation,^2^ the Ribbon and Ribbon TC methods are based on the advanced ‘ribbon-constrained’ technique, which makes use of polyhedra – one polyhedron per vertex, constructed from the vertex’s neighbors on the pial and white matter surfaces. The amount of overlap between the polyhedron and any nearby voxels serves to determine how much these voxels contribute to the data that is finally assigned to the vertex (in the “thin columns” (TC) variant, the polyhedron is constructed slightly differently, in a way which ensures that polyhedra of neighboring vertices do not overlap). In order to isolate the problem, and thus ascertain it really arises from the interpolation of voxel data for surface mapping, we also generated random noise for the vertices in the reconstructed surface directly; the corresponding model is referred to as the “null model”.

**Fig. 2:**
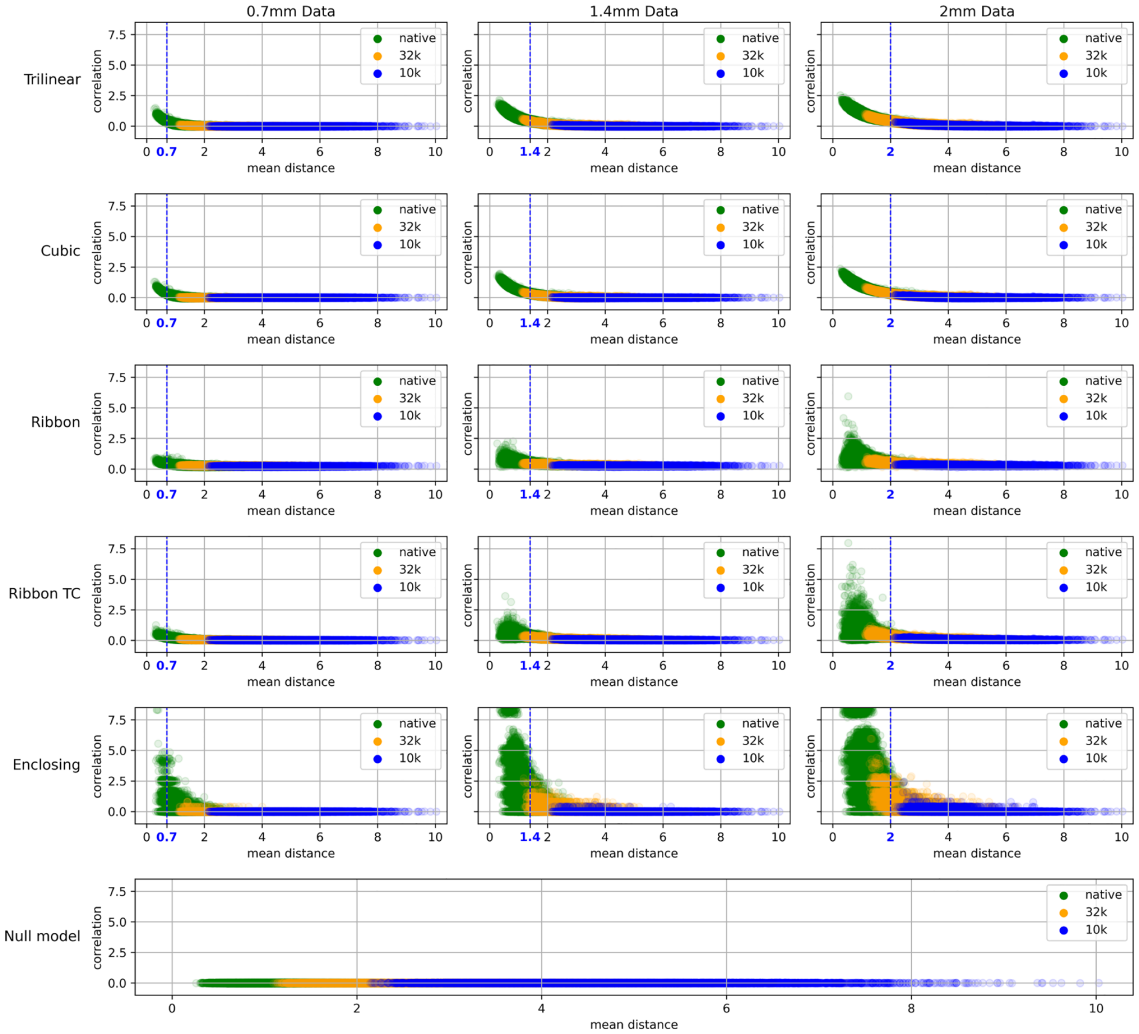
Variation of local correlation with mean inter-vertex distance for various direct mapping approaches and different underlying voxel resolutions (stochastic input in all cases). Data from midline structures were not included. The bottom graph shows what is expected if local correlations are computed for noise timeseries generated directly on a surface mesh (with no volume-to-surface mapping). The vertical dashed (blue) lines indicate the resolution of the underlying volumetric data in one dimension (the voxels are isotropic). As the mean distance between vertices approaches this resolution, geometric effects cause the average local correlation to increase

**Fig. 3:**
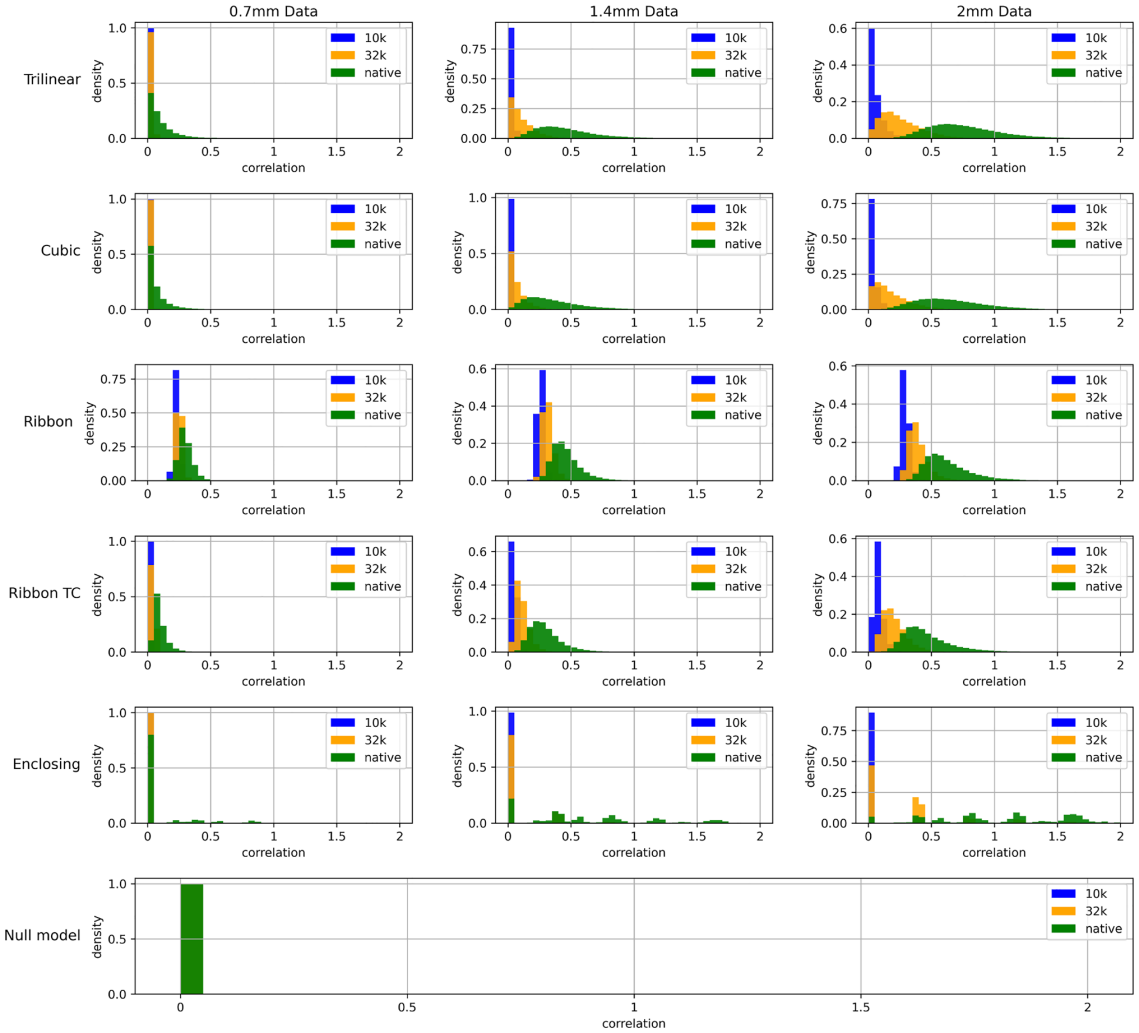
Histograms of surface-based local correlations computed from stochastic volumetric input data. We considered various direct mapping approaches and voxel resolutions. Data from midline structures were excluded

To guarantee a rigorous evaluation of the implications that different volume-to-surface mapping approaches have for any geometric artefacts, we carried out the following:

1. Volume-to-surface mapping of noise timeseries onto the native high-resolution ($\sim 115$k vertices) surface in MNI space, using the five methods detailed above. Then down-sampling the maps to the surface resolution of interest (32k and 10k).^3^ We term this approach the “traditional approach”.
2. Volume-to-surface mapping directly onto surfaces having the required resolution (native, 32k and 10k), using the five approaches. We refer to this as the “direct approach”.
3. Volume-to-surface mapping onto midthickness surface meshes that approximate the resolutions of interest, but that have been modified to reduce the variance in inter-vertex distance (Attene 2010). In this case, we employ the three most basic methods (Trilinear, Cubic and Enclosing).
4. Construction of scatter plots illustrating the relationship between mean distance of neighborhood vertices and local correlations (refer to Fig. 2). We also plotted histograms showing the distribution of local correlations for all the different approaches (Fig. 3).

For the sake of brevity, only the most pertinent results are discussed in the following section. However, all results (including those for the traditional approach) and code are made available for further investigation and scrutiny and may be found at https://osf.io/dxr6f/

## Results

A clear relationship between surface resolution and the size of underlying volumetric elements (voxels) was found. As surface resolution (vertex density) is increased, or voxel data resolution decreased, more surface vertices sample the same voxels from the volume. In both the traditional and direct approaches, this results in artefactual local correlations despite the original, volumetric data being uncorrelated noise. We note that this shortcoming should not be unique to brain data or surface-based processing; upsampling any data should present a similar issue. Figure 2 shows, for the direct approach, that local correlations are approximately zero until the mean distance between neighboring vertices decreases below a certain value (approximately equal to the resolution of the volumetric data). A second feature that stands out in Fig. 2 is the relative poor performance of the Enclosing method, which can be attributed to the fact that this algorithm does not involve any interpolation. As mentioned in the Methodology section, it simply associates with a given vertex the data of the enclosing voxel. The higher the surface resolution in comparison to voxel density, the more likely it is for two vertices to reside within the same voxel and hence be assigned identical data.

## Mitigation

Two main points emerge from the results of our investigations:

1. For local correlation analysis, the resolution of the underlying voxel data should be higher than that of the surface mesh, or at least comparable to it;^4^
2. Any variance in inter-vertex distance on the mesh will create systematic “problematic areas”. As a result, using uniform surface meshes (i.e. meshes with relatively less variation in vertex separation) may mitigate the problem. We have included histograms of inter-vertex distances (mean per neighborhood) for the 10k, 32k and native surfaces (both the regular and uniform meshes) with the supplementary material.

**Fig. 4:**
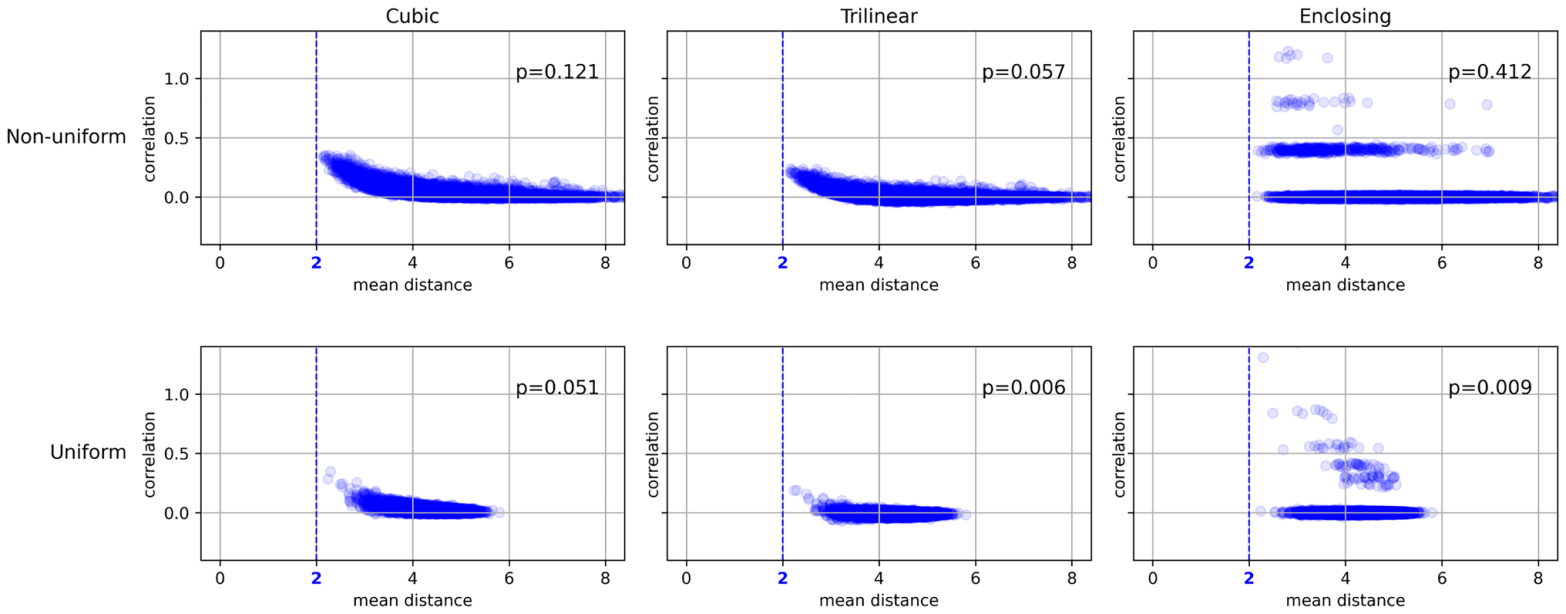
The impact of uniformisation of the mesh. The quantity p in the top right-hand corner of each plot is the $90^\text {th}$ percentile of the distribution of correlations; the fact that it decreases by at least an order of magnitude when using a uniform mesh indicates an overall reduction in neighborhood correlations (and hence in geometric artefacts, since we use stochastic 2 mm data). Surface meshes have a resolution of 10k vertices in all cases

**Fig. 5:**
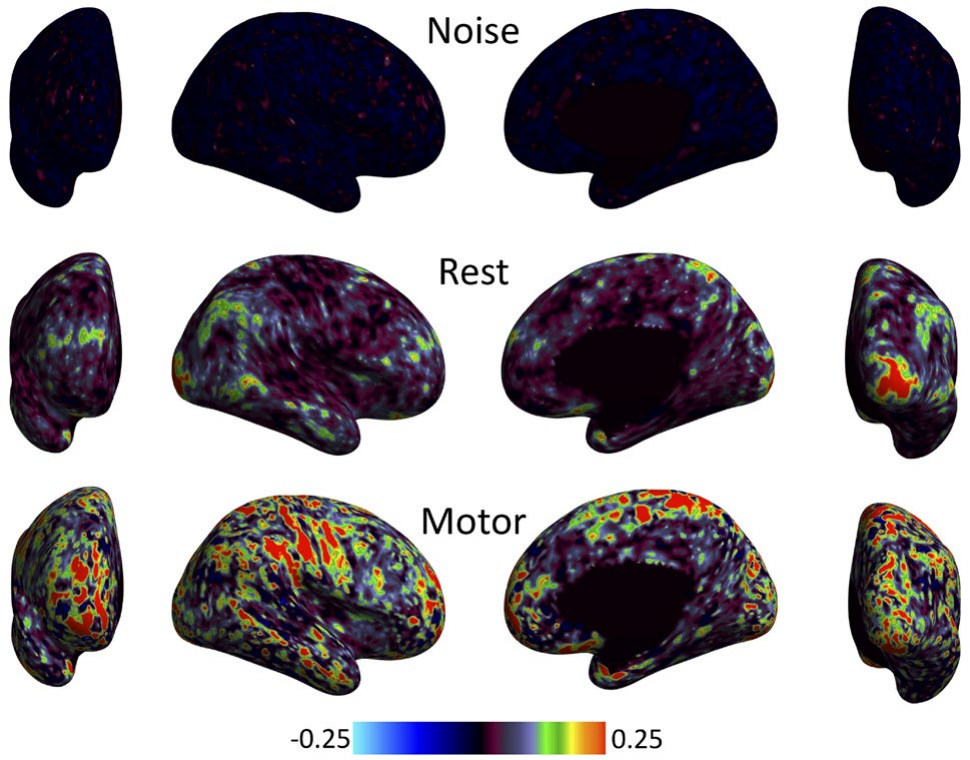
These are the final neighborhood correlation maps, obtained using the direct approach with cubic interpolation and uniform 10k meshes. The difference between Figs. 1 and 5 emerges clearly upon comparison

As can be deduced from Figs. 4 and 5, projecting data at a typical high fMRI resolution (2 mm) onto a 10k *uniform* surface greatly improves local connectivity estimation. Figure 5 (top) shows that the volume-to-surface mapping of stochastic data no longer gives rise to any obvious geometric patterns in local correlations.^5^ Further, additional analysis using real resting-state or task-based fMRI data reveals high local correlations in areas that conform to the expected regions of activity. It is important to note, however, that this approach comes at the cost of (1) decreasing spatial resolution and (2) adding extra steps to the pipeline (needed to regain vertex correspondence to a standard template).

### The hybrid approach

A more theoretically robust approach to decreasing artificial correlations involves mapping each vertex to the corresponding voxel and constructing the associated graph by identifying the closest neighbors in volume space. The local connectivity estimation then proceeds as outlined in the section ‘The problem’, and the result is assigned to the original surface vertex. We used a volume mask to exclude non-brain voxels from the analysis.

**Fig. 6:**
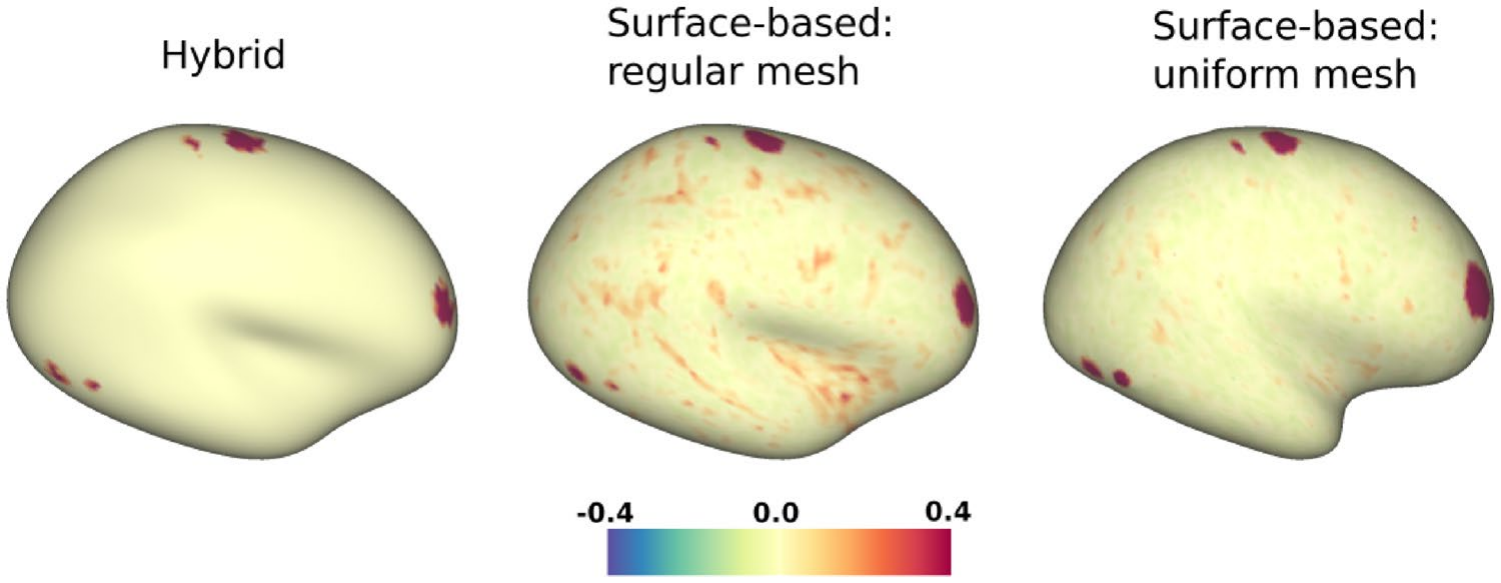
Correlation maps for synthetic data on 10k inflated surfaces. The data were generated as a superposition of task activations and a noise mixture. The figure shows the results obtained with the hybrid method (left), as well as with a surface-based approach on either a regular surface mesh (center) or a uniform mesh (right); in the last two cases, the data were mapped to the surface using cubic spline interpolation before being analysed for correlations (the hybrid method takes volumetric data as input instead)

To test the performance of the algorithm, we generated synthetic fMRI data with the R software package neuRosim (Welvaert et al. 2011). Specifically, we made use of the function simVOLfmri and specified 5 spherical areas of activation. Three lateral areas are clearly visible in Fig. 6: the middle frontal gyrus, the posterior inferior occipital temporal region and the precentral gyrus. We set the signal-to-noise ratio to 4 and the noise to a mixture of the following components: white (rician, with non-centrality parameter equal to 0) (0.05), temporal (0.1), physiological (0.09), task-related (0.05) and spatial (0.7) (the quantities in round brackets indicate the respective weights). The spatial noise was modeled as a Gaussian random field with the full width at half maximum of the kernel set to 4, and a low-frequency drift (0.01) at 0.008 Hz was also included. We put the repetition time TR equal to 2 s.

Fig. 6 shows how the results of the hybrid algorithm compare with those obtained via a wholly surface-based method. It can be seen that the latter (when used on a regular mesh) gives rise to thin strands of correlated vertices which follow the pattern of cortical folds, but that these are completely absent in the case of the hybrid approach. Using a uniform mesh also helps to alleviate the problem. As expected, all three approaches detect the strongest correlations at the activated regions.

## Conclusion

The process of mapping voxel data to a surface mesh can introduce artificial correlations, especially when the geodesic distance between surface vertices is considerably larger than their Euclidean separation. Vertices lying on opposite banks of a sulcus or gyrus are a case in point. Such vertices can easily be contained within the same voxel, despite the fact that the distance between them as measured by a surface metric would exceed the average inter-vertex separation. Consequently, artefacts in functional correlation often trace the pattern of the cortical folding. Mitigation measures include parcellating the brain and using the average time series of each parcel. A second option is to set a threshold distance on the surface and exclude any vertices closer than the threshold from the analysis (Xu et al. 2019). However, both these approaches would preclude the local correlation analysis we focus on in this work, since this involves determining the correlation of a vertex with its immediate neighbors.

By using a variety of voxel and surface resolutions and volume-to-surface mapping methods, we have shown that geometric artefacts may be curtailed (but not necessarily eliminated) by (a) mapping voxel data to a mesh of comparable or lower resolution (and thus reducing the risk of oversampling), (b) ensuring that the inter-vertex distance of the mesh is of relatively low variance, and c) carrying out the analysis in volume space and then mapping the results to the surface. A fourth option would be to construct a correlation map for stochastic data and use this to threshold the results obtained with real data. However, this approach has significant shortcomings—not least the fact that the noise distribution would have to be tuned for different studies—and is not pursued here.

In summary, this study highlights the occurrence of geometric effects in surface-based analyses of local neighborhood connectivity and investigates a number of measures that can alleviate this problem. We caution that any researcher interested in investigating local correlations in brain function should take such effects seriously.

## Data Availability

Data were provided by the Human Connectome Project, WU-Minn Consortium (Principal Investigators: David Van Essen and Kamil Ugurbil; 1U54MH091657) funded by the 16 NIH Institutes and Centers that support the NIH Blueprint for Neuroscience Research; and by the McDonnell Center for Systems Neuroscience at Washington University. We made use of HCP Young Adult data (the S1200 release; see (https://www.humanconnectome.org/study/hcp-young-adult/document/1200-subjects-data-release), processed by the HCP with the following pipelines: Original MSM-Sulc based preprocessing (v3.4.0) Spin Echo Bias Field Prerequisite Files (v3.12.0) MSM-All DeDrifting and Resampling based on MSM-All registration (v3.13.2) Details about the subset of subjects the work is based on and further information about processing/analysis may be obtained from the authors.

## Footnotes

1 In FreeSurfer (http://surfer.nmr.mgh.harvard.edu/), volume-to-surface mapping can be carried out using mri_vol2surf with trilinear interpolation and a projection fraction of 0.5 with the white matter surface.

2 www.humanconnectome.org/software/workbench-command/-volume-to-surface-mapping.

3 The 10k surface was constructed by down-sampling the native mesh registered to MNI space. The code is included with the supplementary material.

4 Surface resolution can be expressed in mm by using the rule of thumb 10k $\rightarrow$ 3.5 mm, 32k $\rightarrow$ 2 mm. In the case of a customized surface, the mean $\mu$ of the inter-vertex distance distribution provides a measure of surface resolution (as long as the standard deviation is relatively small).

5 We verified this by repeating the procedure for 23 other participants included in the data release. In all cases, similar results were obtained (we have made these available as supplementary material).

## Code availability

The code required to recreate the complete analysis is available at https://osf.io/dxr6f/. We also provide a number of additional figures. This project made use of Connectome Workbench (Marcus et al. 2011).
